# Psychological correlates of nonsuicidal self-injury in women with borderline personality disorder: a cross-sectional study to inform mindfulness-based interventions

**DOI:** 10.1186/s12888-025-07740-0

**Published:** 2026-01-16

**Authors:** Szilvia Kresznerits, Ágnes Zinner-Gérecz, Mónika Miklósi, Tamás Szekeres, Dóra Perczel-Forintos

**Affiliations:** 1 Department of Psychiatry and Psychotherapy, Department of Clinical Psychology, Faculty of Medicine, Semmelweis University, Budapest, Hungary; 2 Mental Health Sciences Division, Doctoral School of Semmelweis University, Budapest, Hungary; 3 Department of Developmental and Clinical Child Psychology, Institute of Psychology, Eötvös Loránd University, Budapest, Hungary; 4 Department of Rehabilitation, National Institute of Oncology, Budapest, Hungary

**Keywords:** Borderline personality disorder, Self-injurious behaviour, Mindfulness, Emotional regulation, Impulsive behaviour, Self-compassion, Suicide prevention

## Abstract

**Objective:**

Nonsuicidal self-injury (NSSI) is a significant predictor of suicide, particularly among patients with borderline personality disorder (BPD). This study examined modifiable psychological factors associated with NSSI to generate preliminary insights that may inform future intervention research.

**Methods:**

In a cross-sectional design, 109 female BPD patients completed self-reported measures assessing NSSI behaviours, mindfulness skills, emotion regulation strategies, depressive symptoms, and self-compassion. Correlational analyses were conducted to investigate the relationships among the psychological variables, NSSI frequency, and the number of NSSI methods used. Ordinal logistic general linear models were used to identify potential predictors of NSSI frequency.

**Results:**

Participants reported low levels of self-compassion, mindfulness skills, and self-esteem, alongside high depression, impulsivity, and frequent self-harming behaviour. The key relevant psychological factors associated with more frequent NSSI included increased impulsivity (95% CI: 1.007–1.199), higher scores on the adaptive cognitive-emotional regulation strategies subscale (95% CI: 1.015–1.082), and reduced self-compassion (95% CI: 0.945–0.999). Although mindfulness skills were not directly linked to the frequency of NSSI or the number of methods used, they were moderately correlated with these risk factors.

**Conclusions:**

These findings underscore the importance of targeted interventions for NSSI in BPD patients. Mindfulness-based approaches may reduce suicide risk and improve treatment engagement by addressing deficits in impulsivity, acceptance, and self-compassion.

**Clinical trial number:**

Not applicable (this study was a cross-sectional, observational design and does not meet the criteria for a clinical trial). The project and data are publicly registered at: 10.17605/OSF.IO/ZUR84

**Supplementary Information:**

The online version contains supplementary material available at 10.1186/s12888-025-07740-0.

## Introduction

### Emotion regulation, impulsivity, and mindfulness skills in individuals with borderline personality disorder

Borderline personality disorder (BPD) is a serious, complex, and pervasive mental illness characterised by dysfunctional patterns of instability in self-image, interpersonal relationships, affects, cognitions, and behaviours [1]. Suicide deaths are particularly high in this patient population, with at least 3–6% according to follow-up studies; other studies estimate a 10% suicide rate in BPD patients, with an increased risk of younger age and male sex (Paris, 2019). BPD is also associated with severe functional impairment and a host of dysregulated behaviours that can cause harm to the individual and to the individual’s social environment, such as nonsuicidal self-injury (NSSI), substance use (23–84%), disordered eating (14–53%), and verbal or physical aggression [1–3]. Furthermore, BPD is one of the most common personality disorders, with a point prevalence of 1.6% and a lifetime prevalence of 5.6% [4, 5]. Owing to the frequent and severe crises that characterise the disorder, patients highly utilise the healthcare system; 20% of psychiatric outpatients and 10% of inpatients have a diagnosis of BPD [6]. Given the high personal and societal burden of BPD, effective yet scalable psychotherapeutic interventions are essential. However, long waiting lists due to high demand can often lead to significant deterioration of the condition and increase the risk of suicide [7–9]. Therefore, to mitigate this risk, there is a need for short, evidence-based interventions that address the most critical factors, such as self-harm and emotion regulation difficulties.

In terms of emotional mechanisms, emotion dysregulation is a core trait of BPD and is characterised by heightened emotional vulnerability, insufficient control, intense reactions, and impaired impulse management [10]. These difficulties can even double the risk of NSSI [11, 12]. The *emotional cascade model* [13, 14] emphasises rumination as central to self-harming behaviours in BPD patients. This repetitive worrying intensifies negative emotions, driving individuals to engage in NSSI to cope with or temporarily address overwhelming negative emotions.

In BPD, impulses lead to sudden decisions and actions, especially under stress [15]. According to the *mindfulness deficit theory* [16–18], emotion and impulse control difficulties in BPD patients can be derived from reduced mindfulness skills. BPD patients tend to avoid unpleasant emotions, hindering adaptive coping development. In distressing situations, hypersensitivity and emotion suppression can lead to increased impulsivity and reliance on maladaptive coping mechanisms, such as substance use or self-harm. In contrast, the purpose of mindfulness is to be aware and accepting of experiences, help individuals distance themselves from automatic reactions and respond more flexibly to internal and external stimuli [16, 17].

### NSSI: Triggering and maintaining factors

NSSI refers to the deliberate, direct damage to one’s own body tissue without suicidal intent, carried out for reasons that are not socially or culturally sanctioned [19–21]. NSSI affects 17%-18% of adolescents and 4%-6% of adults. Moreover, among psychiatric inpatients, NSSI rates can reach as high as 80% [22–26]. Self-harming behaviour constitutes one of the diagnostic criteria for BPD, and current empirical studies suggest that NSSI is present in roughly 75–90% of individuals with the disorder [27–29]. Although large-scale epidemiological investigations specifically quantifying NSSI frequency in BPD are still lacking [28], available clinical samples consistently point to a very high prevalence. Importantly, deliberate self-harm is not unique to BPD; it may also emerge in the context of anxiety and depressive disorders, substance use disorders, or trauma-related psychopathology [22, 30–33]. Due to findings revealing its transdiagnostic nature, a distinct diagnostic code was added for NSSI in the DSM-5-TR [34].

NSSI also serves as a predictor of future suicidal behaviour (OR = 4.27, 95% CI = 2.56–7.10) [35]. Repetitive and multimethod NSSI is associated with increased suicide attempts. BPD patients are particularly vulnerable to this pattern, increasing their risk of suicide [32, 36]. In addition to psychological risks, NSSIs can result in medical complications, including infections and tissue damage [37].

Biological factors also contribute significantly to the development of NSSI from a neurobiological perspective [38]. Heritability is estimated to be 40–60%, and gene-environment interactions increase the risk. Childhood adversity and stressors can also influence NSSI risk through various biological pathways. Frontolimbic neural system changes are common in individuals with NSSI, affecting emotion regulation and social processing. However, the role of cognitive control and the reward system in individuals with NSSI is less clear. Individuals with NSSI often show heightened sympathetic nervous system activity, altered cortisol response to stress, reduced pain sensitivity, and neural system changes during NSSI episodes [38].

Psychological vulnerabilities contributing to NSSI include childhood traumatisation, attachment problems, heightened emotional reactivity, low self-esteem, low self-compassion, low anxiety tolerance, and emotion dysregulation, particularly increased rumination [11, 13, 36, 39–43]. Risk factors for NSSI include prior NSSI, cluster B personality disorders, and hopelessness, with social factors also playing a significant role, although to a lesser extent than emotional distress reduction does [32, 44–46].

Psychological models have also been developed to describe the triggering and maintaining factors of NSSI. *The cognitive-emotional model* [47] suggests that emotional reactivity, the mental representation of self-harm, self-representations, and thoughts related to NSSI (i.e., the imagined outcome and the ability to cope) contribute to NSSI as a coping strategy. *The four-function model* [26] outlines four reinforcement processes that maintain NSSI: automatic/social and negative/positive reinforcement. On the basis of the meta-analysis examining this theory, NSSI serves intrapersonal functions, focusing primarily on emotion regulation and reducing negative emotional states. Nevertheless, 5–21% of the tested participants did not use NSSI for emotion regulation. Therefore, treatments should assess all potential maintenance functions [45].

The *cognitive-emotional reactivity model* [48] integrates key elements from the theories mentioned and extends them with insights from the cognitive reactivity model in recurrent depression [49]. In recurrent depression, cognitive reactivity heightens vulnerability after each episode, strengthening associations between different aspects of depression. The cognitive–emotional reactivity model applies this concept to BPD, which is characterised by rapid, cyclical episodes that intensify cognitive and emotional reactivity.

**Fig. 1:**
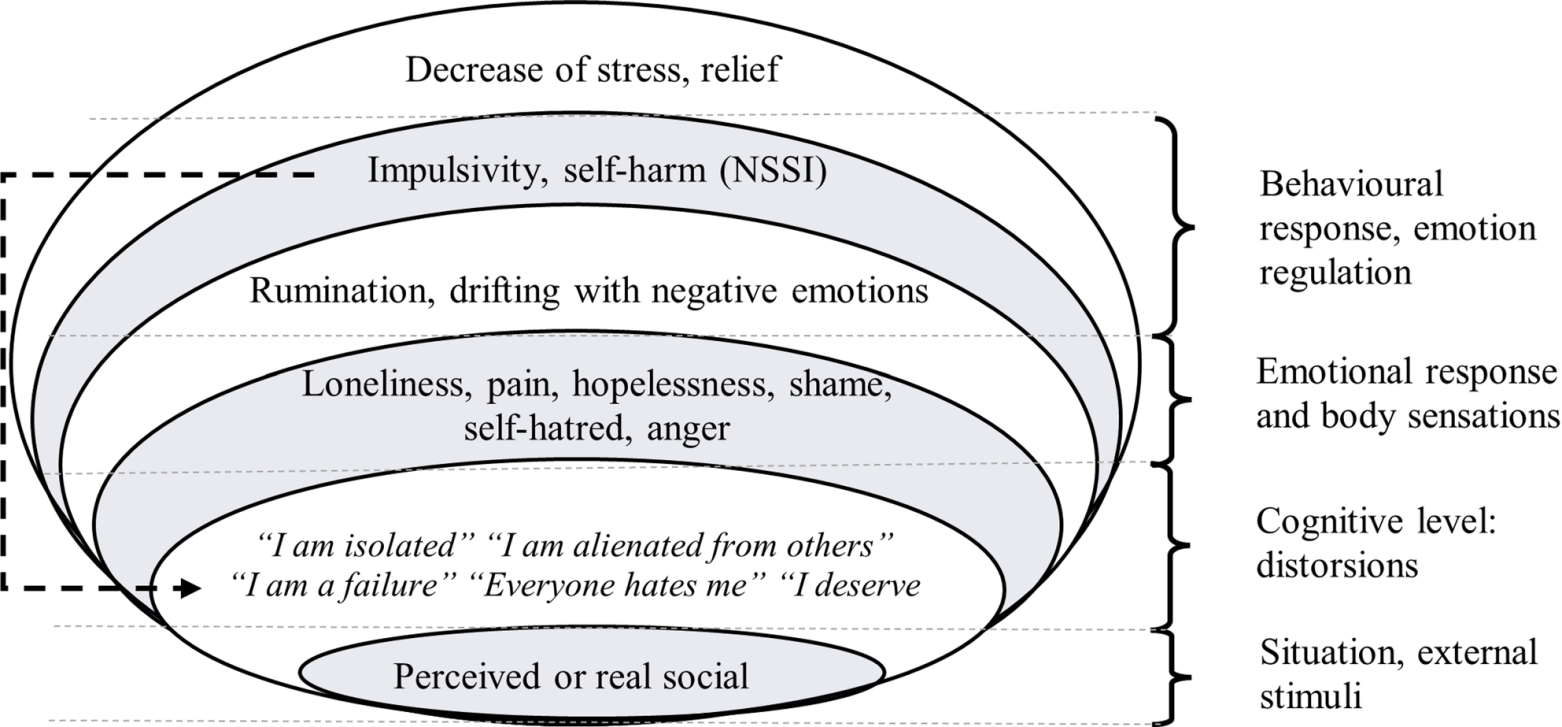
The cognitive-emotional reactivity model of NSSI in BPD

Maladaptive coping strategies such as self-harm reinforce connections between negative thoughts, emotions, and bodily sensations. Triggering events initiate a vortex of negative thoughts and emotions, making it increasingly difficult to identify underlying processes (Fig. 1). Although NSSI may provide immediate emotional relief, it can also deepen self-blame and lead to perceived loss of control. Therefore, therapy aims to teach decentralisation from automatic responses, enabling individuals to break free from this vicious cycle and develop healthier emotional management strategies [48].

### Mindfulness-based practices in BPD and NSSI treatment

Dialectical behaviour therapy (DBT) is a well-established and widely used treatment for BPD that is specifically designed to address core symptoms such as emotion dysregulation, impulsivity, and self-harming behaviours [10, 50]. Although the standard DBT program takes 12 months, adaptations with shorter durations (e.g., 20-week modules) have been developed to improve accessibility in healthcare settings.

Improving mindfulness skills is a foundational module of DBT, enhancing patients’ awareness of internal experiences and reducing automatic, maladaptive responses. The development of mindfulness skills can directly address impulsivity [51, 52] and promote the inhibition of NSSI [53, 54].

Neuroimaging studies suggest that mindfulness practices may alter neural activity in regions associated with the default mode network, emotion regulation, and impulse control, and can reduce overall symptom severity in patients with BPD [55].

In addition to its impact on emotion regulation, mindfulness has also been associated with increased self-compassion, an important protective factor against self-harm. Greater self-compassion has been associated with reduced shame, self-criticism, and thought suppression—mechanisms that often contribute to the maintenance of NSSI [56–60].

### Aims

This study examines the relationships among mindfulness skills, NSSI, and modifiable potentially relevant psychological factors in individuals with BPD. The primary goal is to support the development of targeted mindfulness-based cognitive therapy for NSSI (MBCT-NSSI) by identifying preliminary associations with potential intervention targets for NSSI.

We propose the following hypotheses:

#### (H1)

Mindfulness skills are positively correlated with adaptive emotion regulation, self-esteem, and self-compassion and negatively correlated with maladaptive emotion regulation, depression, hopelessness, impulsivity, and dissociation.

#### (H2)

NSSI severity (i.e., frequency and number of methods used) is positively correlated with maladaptive regulation, depression, hopelessness, impulsivity, and dissociation but negatively correlated with mindfulness skills, adaptive regulation, self-esteem, and self-compassion.

## Method

### Procedures and sample

This study targeted adult BPD outpatients with self-reported NSSI in the past six months. The exclusion criteria included acute suicidal crisis, psychosis, bipolar manic episodes, severe substance use disorder, organic or symptomatic mental disorders, and intellectual disability. The sample size calculation followed the approach proposed by Viechtbauer et al. [61], which provides a general formula for determining sample size in pilot studies to ensure adequate precision in estimating preliminary parameters. Applying this method yielded an estimated required sample size of *N* = 58.7 at 95% confidence with α = 0.05.

Recruitment involved establishing an experimental mindfulness training group for BPD patients who engaged in self-harm. The intervention followed the MBCT protocol adapted for suicide prevention by Williams et al. [49]. Recruitment materials were disseminated across psychiatric departments and outpatient clinics nationwide. Eligible patients who sought individual therapy at our outpatient clinic were also informed about the opportunity to participate in the study and the experimental intervention group.

All the applicants underwent a standard diagnostic procedure in line with the clinic’s protocol, which trained clinical psychologists conducted under the supervision of the last author. National regulations determine diagnoses on the basis of the ICD-10 criteria [62, 63]. The participants completed a semistructured interview about NSSI and a self-report questionnaire (printing or online). The questionnaire contains 18 demographic questions, and the other 160 items assess mindfulness skills, emotion regulation, self-esteem, self-compassion, depression, hopelessness, impulsivity, and dissociation.

A cross-sectional design was employed to examine the relationship between mindfulness and NSSI. Between 1st January 2019 and 30th June 2023, 158 applicants were screened for eligibility. All participants provided written informed consent, and the study received ethical approval. This study was a cross-sectional, observational design and does not meet the criteria for a clinical trial; therefore, no clinical trial number is applicable. The project and anonymised dataset are publicly registered at the Open Science Framework. A total of 120 outpatients (109 women [90.83%] and eleven men [9.16%]) met the inclusion criteria. To control for gender-related bias, only data from female participants were analysed. The final sample comprised 109 female BPD outpatients (M_age_: 27.50 years, SD = 7.85). Although the primary analyses were restricted to women to reduce sample heterogeneity in psychological predictors, we conducted additional sensitivity analyses including the 11 male participants, and the overall pattern of results remained unchanged (see Supplementary Materials).

Education levels: 9.2% (*N* = 10) had basic education, 55.0% (*N* = 60) had secondary education, and 35.8% (*N* = 39) had a degree in higher education. Employment status: 28.4% (*N* = 31) were students, 48.6% (*N* = 53) were employed or self-employed, and 22.9% (*N* = 25) were unemployed or had other passive employment statuses. Comorbidities: A total of 74.31% (*N* = 81) had at least one comorbid diagnosis beyond BPD, and 22.02% (*N* = 24) had at least two additional diagnoses (Table 1).

**Table 1:** Prevalence of comorbid disorders in the BPD sample (*N* = 109)

Comorbid diagnosis (ICD-10)		%	∑%
Depression	Bipolar (F31.3, F31.6, F31.8)	12.84	40.37
	Unipolar (F32.0, F32.1, F32.2, F32.8)	18.35	
	Recurrent/persistent (F33.0, F33.1, F34.8)	9.17	
Neurotic, stress-related, and somatoform disorders	Mixed anxiety and depressive disorder (F41.2)	21.10	40,37
	Anxiety disorders (F40.0-F41.8, without F41.2)	11.01	
	Obsessive-compulsive disorder (F42.0-F42.2)	1.83	
	Posttraumatic stress disorder (F43.1)	6.42	
Eating disorders (F50.0, F50.2, F50.8)		11.01	11.01
Comorbid personality disorder		11.01	11.01
Other (F19.1 in remission, F51.0, F63.8, F84.8)		4.59	4.59

The manuscript text was edited via Grammarly (Grammarly Inc., San Francisco, CA) for grammar and language clarity. All content and interpretations are the original work of the authors.

### Measures

The following self-administered questionnaires were included in the statistical data analysis:

- **The general data sheet** captured demographic data, psychiatric history, and details of suicide attempts.
- **Rosenberg Self-Esteem Scale (RSES)**: A 10-item measure of global self-worth rated on a 4-point Likert scale [64, 65].
- **Five-Facet Mindfulness Questionnaire (FFMQ)**: A 39-item measure of mindfulness traits across five subscales: *observing*, *describing*, *acting with awareness*, *nonjudging inner experience*, and *nonreactivity* [60]. The adapted Hungarian questionnaire version [66] is under standardisation.
- **Beck Depression Inventory – Shortened version (BDI-S)**: A 9-item measure of depression severity [67, 68].
- **Beck Hopelessness Scale – Shortened version (BHS-S)**: A 4-item abbreviated scale of the original Beck Hopelessness Scale measuring hopelessness [69, 70].
- **Barratt Impulsivity Scale-Shortened (BIS-8-S)**: An 8-item self-report scale measuring impulsivity on a four-point Likert scale [71, 72].
- **Dissociative Experiences Scale (DES)**: A 28-item self-report questionnaire measuring the frequency of dissociative experiences, with responses ranging from 0 to 100 [73, 74].
- **Cognitive Emotion-Regulation Questionnaire (CERQ)**: A 36-item self-report measure to evaluate cognitive strategies in emotion regulation [75] with nine subscales [76, 77]. *Self-blame*, *rumination*, *catastrophizing*, and *blaming others* represent maladaptive strategies, *whereas refocusing on planning*, *positive reappraisal*, *putting into perspective*, *positive refocusing*, and *acceptance* represent adaptive strategies.
- **Self-Compassion Scale (SCS)**: A 26-item self-report instrument with three subdimensions of self-concept: *self-judgment* vs. *self-kindness*, *isolation* vs. *common humanity*, and *overidentification* vs. *mindfulness* [78, 79].

**Fig. 2:**
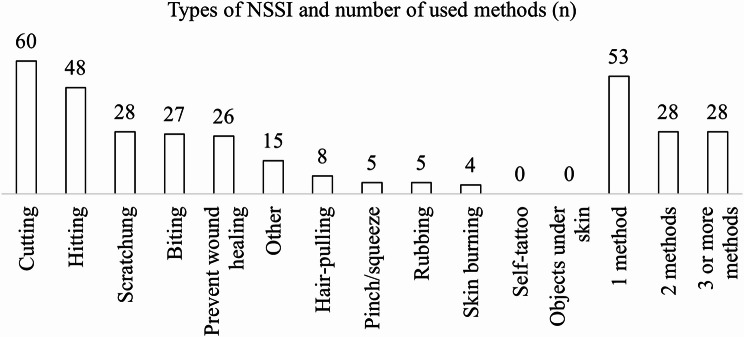
Descriptive statistics of the NSSI methods used. Notes. n = number of patients who used the specified NSSI method. N_total participants_ = 109

NSSI frequency and severity (types and methods) were assessed via a semistructured interview. The interview questions included “*Have you ever deliberately harmed yourself without intending to die?” “Have you done so in the past six months?”* and “How many times or how often?” Participants who were unsure or unfamiliar with the terminology were provided with examples (e.g., cutting, burning). If one form of NSSI was identified, the others were queried systematically. If one type occurred, we also asked about other types based on the list shown in Fig. 2, including two additional categories (inserting objects under the skin or nails and tattooing), which did not occur in this sample. Following the semi-structured interviews, NSSI frequency responses were assigned to predefined ordinal categories (1 = *1–3 times per year*, 2 = *approximately monthly*, 3 = *approximately weekly*, 4 = *daily or more frequently*). This categorisation was part of the coding process and did not influence the assessment procedure or participant inclusion.

### Data analyses

Data analysis was conducted with IBM SPSS Statistics 28©. Missing values were excluded from the study. Missing data ranged from 0% to 3% across variables. A significance level (α) of 0.05 was used. Pearson’s correlation was employed to examine the relationships between continuous variables (e.g., clinical scales and number of NSSI methods). Spearman’s rank-order correlation was applied to assess associations between ordinal NSSI frequency categories and clinical variables. Ordinal logistic general linear models (GLMs) were used to identify predictors of NSSI frequency.

## Results

### Descriptive analysis of the clinical and psychometric scales

Table 2 presents the internal consistency and descriptive statistics of the administered psychometric instruments. All scales demonstrated acceptable reliability for early-stage research (Cronbach’s α > 0.70), which is consistent with the guidelines of Nunnally and Bernstein [80]. However, two FFMQ subscales (*observing* and *nonreactivity*) and the BIS-8-S presented Cronbach’s alpha values less than 0.80, indicating suboptimal reliability for applied research settings. Based on the confirmatory factor analysis (extraction method: maximum likelihood, rotation method: varimax with Kaiser normalisation), two items from the adaptive subscale of the CERQ were excluded from the analysis. *Item 20* loaded more strongly on the maladaptive scale than on the adaptive scale, whereas *item 23* had similar loadings on both factors (0.387 and 0.329). The revised scale is referred to as CERQ_ad*.

**Table 2:** Internal reliabilities and descriptive statistics of the clinical and psychometric scales

Scales	Cronbach’s α	M	SE	Range	Reference Values (Normative Means / Clinical Cut-offs)
RSES	0.855	10.128	0.535	0–30	> 15 (65)
FFMQ_o	0.701	25.835	0.538	8–40	
FFMQ_d	0.906	23.917	0.718	8–40	
FFMQ_a	0.824	20.724	0.580	8–40	
FFMQ_nj	0.833	20.239	0.623	8–40	
FFMQ_nr	0.714	14.651	0.416	7–35	
FFMQ	0.825	105.367	1.574	39–195	*M* = 133.80, *SD* = 21.58 (72)
CERQ_ad	0.896	51.248	1.343	20–100	*M* = 64.57, *SD* = 10.33 (77)
CERQ_ad*	0.910				
CERQ_mad	0.863	50.505	1.092	16–90	*M* = 39.04, *SD* = 8.01 (77)
BDI-S	0.801	22.055	0.499	9–36	< 19 (67)
BHS-S	0.884	10.275	0.356	4–16	< 9 (70).
BIS-8-S	0.757	20.679	0.405	8–32	*M* = 15.46, *SD* = 4.98 (72)
DES	0.930	772.018	43.561	0–2800	
SCS	0.898	52.576	1.444	26–130	*M* = 70.31, *SD* = 12.11 (79)

Notes: *N* = 109. Reference Values (normative means / clinical cut-offs) were based on nationally standardised values for the general population, where such data were available. RSES = Rosenberg Self-Esteem Scale, FFMQ = Five-Facet Mindfulness Questionnaire, FFMQ_o = FFMQ observing subscale, FFMQ_d = FFMQ describing subscale, FFMQ_a = FFMQ acting with awareness subscale, FFMQ_nj = FFMQ nonjudging to inner experience subscale, FFMQ_nr = FFMQ nonreactivity to inner experience subscale, CERQ = Cognitive Emotion Regulation Questionnaire, CERQ_ad = CERQ adaptive strategies subscale, CERQ_mad = CERQ maladaptive strategies subscale, CERQ_ad* = modified CERQ adaptive subscale, excluding items 20 and 23, BDI-S = Beck Depression Inventory Shortened, BHS-S = Beck Hopelessness Inventory Shortened, BIS-8-S = Barratt Impulsivity Scale Shortened, DES = Dissociative Experience Scale, SCS = Self-Compassion Scale

The participants, on average, demonstrated low self-esteem, self-compassion, and mindfulness skills. The mean depression and hopelessness scores exceeded the values typical for the general population, and the impulsivity levels were also elevated. In terms of emotion regulation, the scores for adaptive strategies were lower than the Hungarian normative values, whereas maladaptive strategy scores were noticeably higher (Table 2).

### Correlation of mindfulness skills with clinical and psychometric variables

The total mindfulness score (FFMQ) was positively correlated with protective psychological factors—self-esteem, self-compassion, and adaptive emotion regulation strategies. In contrast, moderate negative correlations emerged between total mindfulness and depression, hopelessness, impulsivity, and dissociation. Although the correlations with specific emotion regulation strategies were weak, they aligned with the theoretical expectations (see Table 3).

Among the FFMQ subscales, *acting with awareness*, *nonjudging inner experience*, and *nonreactivity to inner experience* demonstrated the strongest associations with other psychological constructs. Specifically, *acting with awareness* showed moderate negative correlations with impulsivity, depression, and dissociation. *Nonjudging* was positively correlated with self-esteem and self-compassion and negatively correlated with maladaptive emotion regulation, depression, and dissociation. Finally, *nonreactivity* was strongly positively correlated with self-compassion and moderately negatively correlated with depression (Table 3).

**Table 3:** Pearson’s correlations between FFMQ subscales and other psychometric scales in BPD outpatients

Scale		SCS	RSES	CERQ_ad*	CERQ_mad	BDI-S	BHS-S	BIS-8-S	DES
FFMQ	r	**0.402**	**0.504**	**0.291**	**-0.228**	**-0.425**	**-0.339**	**-0.478**	**-0.369**
	p	**< 0.001**	**< 0.001**	**0.002**	**0.017**	**< 0.001**	**< 0.001**	**< 0.001**	**< 0.001**
FFMQ observing	r	0.090	0.092	0.176	0.105	0.055	-0.071	-0.019	**0.206**
	p	0.360	0.342	0.068	0.278	0.568	0.466	0.848	**0.032**
FFMQ describing	r	< 0.001	0.202	0.077	0.105	-0.155	**-0.191**	-0.086	**-0.276**
	p	0.999	0.035	0.428	0.275	0.107	**0.046**	0.376	**0.004**
FFMQ acting with awareness	r	0.110	**0.387**	0.059	**-0.252**	**-0.326**	**-0.194**	**-0.435**	**-0.414**
	p	0.260	**< 0.001**	0.543	**0.008**	**0.001**	**0.043**	**< 0.001**	**< 0.001**
FFMQ nonjudging	r	**0.464**	**0.425**	0.172	**-0.376**	**-0.398**	**-0.260**	-0.162	**-0.352**
	p	**< 0.001**	**< 0.001**	0.074	**< 0.001**	**< 0.001**	**0.006**	0.093	**< 0.001**
FFMQ nonreactivity	r	**0.530**	**0.262**	**0.403**	**-0.263**	**-0.359**	**-0.199**	**-0.300**	-0.081
	p	**< 0.001**	**0.006**	**< 0.001**	**0.006**	**< 0.001**	**0.038**	**0.002**	0.403

Notes: *N* = 109, FFMQ = Five-Facet Mindfulness Questionnaire, SCS = Self-Compassion Scale, RSES = Rosenberg Self-Esteem Scale, CERQ = Cognitive Emotion Regulation Questionnaire, CERQ_ad*=CERQ modified adaptive subscale, without items 20 and 23, CERQ_mad = CERQ maladaptive strategies subscale, BDI-S = Beck Depression Inventory Shortened, BHS-S = Beck Hopelessness Inventory Shortened, BIS-8 = Barratt Impulsivity Scale Shortened, DES = Dissociative Experience Scale. Bolded correlations = significant

### Nonsuicidal self-injury (NSSI)

Figure 2 illustrates the frequency of NSSI methods reported by participants. Self-hitting and skin-cutting were the most common types of NSSI, and 51.37% of the patients used multimethod NSSI.

In terms of frequency, 13.8% of the patients engaged in NSSI *1–3 times per year*, 39.4% *monthly*, 32.1% *weekly*, and 14.7% *at least daily*.

None of the assessed psychological variables (e.g. mindfulness skills, dissociation, impulsivity, self-compassion, self-esteem, depressive symptoms, or emotion regulation strategies) showed significant bivariate correlations with either NSSI frequency (Spearman’s ρ) or with the number of NSSI methods (Pearson’s r).

### Predictive models of NSSI frequency

To identify potential predictors of NSSI frequency, two ordinal logistic general linear models (GLMs) were constructed. In both initial models, the outcome variable was NSSI frequency, with *1–3 events per year* used as the reference category. In the first model, predictor variables included the FFMQ total score; CERQ_mad and CERQ modified adaptive strategies subscale; and scores from the BDI-S, BIS-8-S, SCS, and DES. A multicollinearity check was used before the analyses. To minimise multicollinearity, the Beck Hopelessness Scale (BHS-S) was excluded from the model because it showed an extremely strong correlation with the BDI (*r* = .696). There was no collinearity or multicollinearity between the remaining variables (*r* < .528 and VIF = 1.128–1.746 in all cases).

After stepwise model refinement, three predictors were retained on the basis of statistical significance (see Table 4). While the model exhibited weak explanatory power, it still demonstrated a significant improvement in fit over the null model (Χ^2^ (3) = 12.529, *p* = .006).

**Table 4:** Parameter estimates in the ordinal GLM of NSSI frequency

Parameter	OR (Exp(B))	*p*	95% Wald CI
BIS-8-S	1.099	0.035	1.007–1.199
CERQ_ad*	1.048	0.004	1.015–1.082
SCS	0.972	0.043	0.945–0.999

Notes: BIS-8-S = Barratt Impulsivity Scale Shortened; SCS = Self-Compassion Scale; CERQ_ad*=Cognitive Emotion Regulation Questionnaire modified adaptive subscale, without items 20 and 23. Model: Χ2 (3) = 12.529, *p* = .006

In the second model, predictor variables included age; the number of comorbid diagnoses; and dummy-coded variables for sex, history of suicide attempts (yes vs. no), and family history of suicide (yes vs. no). There was no collinearity or multicollinearity among the variables (*r* < .238 and VIF = 1.014–1.113 in all cases). No significant model could be established with these predictors.

## Discussion

### Interpretation of results

This cross-sectional study aimed to explore the complex relationships among mindfulness, emotion regulation, and NSSI among patients with BPD to find factors that may be relevant for intervention targets in a time-limited MBCT-based intervention. All participants were female outpatients enrolled in an experimental MBCT group at a nationwide specialist clinic.

Over half of the sample reported engaging in multimethod NSSI, and 45.3% reported self-harming at least weekly, which is consistent with prior findings [32, 36]. In addition to BPD, most patients have comorbid mood or personality disorders. Self-reported data revealed low levels of self-esteem, self-compassion, and mindfulness skills, alongside elevated levels of depression, hopelessness, and impulsivity. These findings align with previous studies investigating risk factors associated with NSSI [11, 32, 44, 46].

Consistent with mindfulness deficit theory [16–18], mindfulness skills were notably diminished across all FFMQ subscales, with the most significant deficit observed in the *nonjudging* facet.

Our first hypothesis (H1) was confirmed: higher levels of mindfulness were associated with higher self-esteem, self-compassion, and adaptive emotion regulation and inversely related to depression, hopelessness, impulsivity, and dissociation. These findings align with earlier research supporting the protective role of mindfulness against psychological distress [51, 52, 55, 60].

Our second hypothesis (H2)—that the two indicators of NSSI severity (frequency and number of methods) are significantly associated with clinical and psychometric variables—was not supported. None of the bivariate correlations between NSSI and the assessed psychological factors reached statistical significance. This lack of association suggests that, within this sample, NSSI behaviour may not be linearly related to commonly assessed psychological constructs such as mindfulness deficits, dissociation, impulsivity, or emotion regulation difficulties. These null findings should be interpreted with caution, as the absence of statistically significant associations does not necessarily indicate the absence of underlying relationships; it may reflect methodological or sample-related limitations. On the other hand, it may reflect the complexity of NSSI in individuals with BPD, indicating that its function or severity may be influenced by other, unmeasured variables or dynamic, context-specific factors not captured in static self-report measures.

### Unexpected findings

Despite a lack of bivariate associations, an ordinal logistic GLM revealed a statistically significant model for potentially relevant psychological characteristics associated with NSSI frequency. Self-compassion emerged as a potential protective factor, and impulsivity was associated with more frequent NSSI, which is consistent with prior findings [54]. Unexpectedly, however, higher scores on the adaptive cognitive emotional strategies subscale were also associated with more frequent NSSI as a potential risk factor. This counterintuitive result may reflect interpretive or measurement challenges and warrants further investigation.

#### Context-sensitive implementation

Prior research has highlighted that the putative adaptiveness of emotion regulation strategies depends on their type and flexible, context-sensitive implementation. Types of emotions and levels of stress can turn a putatively adaptive strategy into maladaptive coping, or they can also inhibit the practical application of an adaptive strategy known at a cognitive level [81–83]. Moreover, clinical populations may differ fundamentally from nonclinical populations in how such strategies function [84]. For example, items from the CERQ’s putting into perspective subscale (e.g., *“I think that other people go through much worse experiences”*) may unintentionally encourage suppression or self-invalidation in BPD, rather than resilience.

#### Polyregulation and functional sequences

There is also growing evidence of polyregulation and the dynamic use of complex strategies during emotional regulation [85, 86]. In this context, NSSI might serve as an acute emotion-suppression mechanism that temporarily enables cognitive regulation strategies. Thus, the correlation may reflect a reversed pathway—adaptive strategy use may follow NSSI but not precede it.

#### Self-reflective and semantic distortions in BPD

Another possible explanation involves BPD-specific cognitive‒affective biases. Individuals with BPD often have a fragmented self-concept and heightened sensitivity to evaluative language [87–89]. They tend to exhibit negative self-referential processing biases, interpreting even neutral or positive self-statements through a distorted lens, further undermining emotional stability [88, 89]. This may cause them to interpret positively valenced self-reported items (e.g., *“best*,*” “pleasant*,*” “unpleasant”*) in distorted ways, leading to paradoxically high scores on adaptive subscales that reflect identity disturbance or vulnerability, not coping skills. Impaired identity is associated with increased rumination and reduced self-control [87], all of which can lead to increased emotional distress and a greater reliance on NSSI as a maladaptive coping mechanism.

An alternative explanation involves the BPD population’s heightened susceptibility to evaluative language and impaired self-reflection. Individuals with BPD are often characterised by a fragmented and incoherent self-concept, which increases sensitivity to evaluative phrasing and reduces self-reflective capacity [87–89]. They tend to exhibit negative self-referential processing biases, interpreting even neutral or positive self-statements through a distorted lens, further undermining emotional stability [88, 89]. These tendencies may help explain the unexpected appearance of adaptive emotion regulation strategies as risk factors in the model. When individuals with BPD encounter evaluative self-report items, such as those in the CERQ subscales (e.g., “pleasant,” “best,” “bad,” and “unpleasant” about self or behaviours), their elevated scores on some “adaptive” subscales may paradoxically reflect identity disturbance and heightened vulnerability rather than true coping ability. More impaired identity is associated with intensified ruminative processing, diminished self-control, and reduced identity integration [87], all of which can lead to increased emotional distress and a greater reliance on NSSI as a maladaptive coping mechanism.

Future research should explore these interpretations and incorporate qualitative methods (e.g., three-step test interviews [90]) to better understand how individuals interpret these items. It is essential to determine whether high scores on these adaptive subscales reflect genuine coping strategies or maladaptive responses distorted by BPD-specific cognitive-affective biases.

Although mindfulness was not a direct potential predictor of NSSI frequency in the ordinal logistic GLM, potentially relevant psychological factors in the model (self-compassion, acceptance, impulsivity) were all closely associated with mindfulness. This finding is consistent with studies suggesting an indirect role of mindfulness through mediating psychological processes [54]. Furthermore, it is also consistent with broader models of NSSI, including the four functions [26], the cognitive-emotional [47], and the cognitive-emotional reactivity [48] models. However, this finding contrasts with that of Per [58], who identified the direct effects of *nonjudging* and *acting with awareness* on NSSI engagement.

### Clinical implications

From a clinical standpoint, these findings highlight the importance of assessing not only the self-reported use of cognitive emotion regulation strategies but also their depth, flexibility, and functional implementation. The apparent high use of adaptive strategies may mask the limited capacity for emotionally grounded application, particularly in individuals with BPD. This underscores the need to move beyond cognitive instruction. Emotional regulation skills must be developed through experiential learning, generalised across emotional intensities and contexts, and integrated at the cognitive, affective, and bodily levels.

Therapeutic approaches such as DBT, mentalization-based therapy (MBT), and MBCT already incorporate these elements and may be particularly suitable for addressing this regulatory mismatch.

Finally, our results also support the relevance of loving-kindness meditation for individuals with BPD. Previous studies [56–59] have shown that this practice enhances self-compassion as an essential protective factor against NSSI, as identified in our results.

### Strengths and limitations

A primary limitation of this study is the cross-sectional design, which restricts inferences to preliminary associations. Consequently, the findings may guide hypotheses for future longitudinal or intervention studies, but cannot directly inform therapy development.

Another limitation was the use of an interview-based method for assessing NSSI rather than a validated self-report measure due to the lack of such instruments in the local language at the time of data collection. Although this approach provides structured insights, including standardised tools in future research would enhance reliability and comparability.

Given the exploratory nature of the study and the theoretical aim of identifying potential targets for mindfulness-based interventions, we did not apply corrections for multiple comparisons. This increases the risk of Type I errors, and results should be interpreted accordingly.

Additionally, concerns around the interpretive validity of certain CERQ subscales, particularly in the BPD population, highlight the need for mixed-methods research. The incorporation of qualitative interviews, such as the three-step test interview [78], may help contextualise patients’ interpretations of adaptive vs. maladaptive coping strategies.

Although the main analyses were limited to women due to sample homogeneity, supplementary sensitivity analyses including male participants yielded comparable results, suggesting that the findings are robust across sex. However, trend-level differences emerged, which warrant further investigation in larger samples.

Despite these limitations, the study has several strengths. The clinical sample reflects real-world treatment-seeking BPD patients across multiple institutions, enhancing generalizability. The relatively large clinical sample and multimethod assessment approach—including both self-report scales and clinical interviews—provided a comprehensive understanding of psychological functioning in this population.

### Summary

This study explored the relationships among mindfulness, NSSI, and emotion regulation in BPD patients to lay the groundwork for a time-limited, targeted MBCT-based intervention. The participants exhibited low self-compassion, mindfulness, and self-esteem, alongside high levels of impulsivity, depression, and frequent NSSI, thus highlighting a profile of elevated vulnerability.

The findings identified low self-compassion, reduced acceptance, and high impulsivity as key risk factors for frequent NSSI. Although mindfulness was not a direct predictor of NSSI, its moderate associations with protective and risk factors suggest that it could indirectly influence NSSI behavior.

These insights support the need for interventions that strengthen self-compassion and acceptance while addressing impulsivity and potentially maladaptive interpretations of otherwise adaptive strategies. Enhancing mindfulness—especially *nonjudging* and nonreactive mindfulness—may improve emotional regulation and reduce NSSI vulnerability.

Overall, this study highlights the psychological mechanisms most relevant for a brief, targeted MBCT intervention. While further research is essential, especially with more refined measures and qualitative insights, prioritising psychological targets such as impulsivity and self-harm vulnerability may lead to more effective, scalable interventions for BPD, an urgent need in the face of limited healthcare resources and elevated suicide risk.

## Supplementary Information

Below is the link to the electronic supplementary material.

Supplementary Material 1

## Data Availability

The deidentified dataset and supporting documentation are openly available via the Open Science Framework (OSF): https://osf.io/29qn6.
