# Supplementary material for: Psychological correlates of nonsuicidal self-injury in women with borderline personality disorder: a cross-sectional study to inform mindfulness-based interventions
Source: BMC Psychiatry. 2026 Jan 16;26:144. doi: 10.1186/s12888-025-07740-0 (PMC12895986; doi:10.1186/s12888-025-07740-0)
Supplement: Supplementary file 1 — Supplementary Material 1 [file 12888_2025_7740_MOESM1_ESM.docx]

**Supplementary materials**

Table S1a. Pearson’s correlations between FFMQ subscales and other psychometric scales in BPD outpatients (N = 109, only women patients with BPD)

| Scale |  | SCS | RSES | CERQ_ad* | CERQ_mad | BDI-S | BHS-S | BIS-8-S | DES |
| --- | --- | --- | --- | --- | --- | --- | --- | --- | --- |
| FFMQ | r | **0.402** | **0.504** | **0.291** | **-0.228** | **-0.425** | **-0.339** | **-0.478** | **-0.369** |
|  | p | **<0.001** | **<0.001** | **0.002** | **0.017** | **<0.001** | **<0.001** | **<0.001** | **<0.001** |
| FFMQ observing | r | 0.090 | 0.092 | 0.176 | 0.105 | 0.055 | -0.071 | -0.019 | **0.206** |
|  | p | 0.360 | 0.342 | 0.068 | 0.278 | 0.568 | 0.466 | 0.848 | **0.032** |
| FFMQ describing | r | <0.001 | 0.202 | 0.077 | 0.105 | -0.155 | **-0.191** | -0.086 | **-0.276** |
|  | p | 0.999 | 0.035 | 0.428 | 0.275 | 0.107 | **0.046** | 0.376 | **0.004** |
| FFMQ acting with awareness | r | 0.110 | **0.387** | 0.059 | **-0.252** | **-0.326** | **-0.194** | **-0.435** | **-0.414** |
|  | p | 0.260 | **<0.001** | 0.543 | **0.008** | **0.001** | **0.043** | **<0.001** | **<0.001** |
| FFMQ nonjudging | r | **0.464** | **0.425** | 0.172 | **-0.376** | **-0.398** | **-0.260** | -0.162 | **-0.352** |
|  | p | **<0.001** | **<0.001** | 0.074 | **<0.001** | **<0.001** | **0.006** | 0.093 | **<0.001** |
| FFMQ nonreactivity | r | **0.530** | **0.262** | **0.403** | **-0.263** | **-0.359** | **-0.199** | **-0.300** | -0.081 |
|  | p | **<0.001** | **0.006** | **<0.001** | **0.006** | **<0.001** | **0.038** | **0.002** | 0.403 |

**Notes.** FFMQ = Five-Facet Mindfulness Questionnaire, SCS=Self-Compassion Scale, RSES = Rosenberg Self-Esteem Scale, CERQ = Cognitive Emotion Regulation Questionnaire, CERQ_ad*=CERQ modified adaptive subscale, without items 20 and 23, CERQ_mad = CERQ maladaptive strategies subscale, BDI-S=Beck Depression Inventory Shortened, BHS-S= Beck Hopelessness Inventory Shortened, BIS-8=Barratt Impulsivity Scale Shortened, DES=Dissociative Experience Scale.

Bolded correlations indicate statistical significance at p < 0.05.

Table S1b. Pearson’s correlations between FFMQ subscales and other psychometric scales in BPD outpatients (N = 120, including men patients with BPD)

| Scale |  | SCS | RSES | CERQ_ad* | CERQ_mad | BDI-S | BHS-S | BIS-8-S | DES |
| --- | --- | --- | --- | --- | --- | --- | --- | --- | --- |
| FFMQ | r | **0.403** | **0.464** | **0.281** | **-0.233** | **-0.390** | **-0.315** | **-0.320** | **-0.339** |
|  | p | **0.000** | **<0.001** | **0.002** | **0.010** | **<0.001** | **<0.001** | **<0.001** | **<0.001** |
| FFMQ observing | r | 0.124 | 0.030 | **0.202** | 0.098 | 0.111 | 0.005 | -0.006 | **0.277** |
|  | p | 0.184 | 0.744 | **0.027** | 0.288 | 0.228 | 0.959 | 0.952 | **0.002** |
| FFMQ describing | r | 0.004 | **0.203** | 0.05 | 0.120 | -0.156 | -0.179 | -0.140 | **-0.283** |
|  | p | 0.967 | **0.026** | 0.590 | 0.194 | 0.089 | 0.051 | 0.128 | **0.002** |
| FFMQ acting with awareness | r | 0.105 | **0.358** | 0.036 | **-0.248** | **-0.297** | **-0.191** | **-0.438** | **-0.391** |
|  | p | 0.258 | **<0.001** | 0.7 | **0.006** | **0.001** | **0.036** | **<0.001** | **<0.001** |
| FFMQ nonjudging | r | **0.410** | **0.403** | **0.134** | **-0.399** | **-0.381** | **-0.294** | -0.158 | **-0.364** |
|  | p | **<0.001** | **<0.001** | **0.44** | **<0.001** | **<0.001** | **0.001** | 0.084 | **<0.001** |
| FFMQ nonreactivity | r | **0.532** | **0.246** | **0.43** | **-0.262** | **-0.349** | -0.171 | -0.110 | -0.070 |
|  | p | **<0.001** | **0.007** | **<0.001** | **0.004** | **<0.001** | 0.061 | 0.232 | 0.447 |

**Notes.** FFMQ = Five-Facet Mindfulness Questionnaire, SCS=Self-Compassion Scale, RSES = Rosenberg Self-Esteem Scale, CERQ = Cognitive Emotion Regulation Questionnaire, CERQ_ad*=CERQ modified adaptive subscale, without items 20 and 23, CERQ_mad = CERQ maladaptive strategies subscale, BDI-S=Beck Depression Inventory Shortened, BHS-S= Beck Hopelessness Inventory Shortened, BIS-8=Barratt Impulsivity Scale Shortened, DES=Dissociative Experience Scale.

Bolded correlations indicate statistical significance at p < 0.05.

Table S2a. Parameter estimates in the ordinal logistic GLM of NSSI frequency (N = 109, only women patients with BPD)

| Parameter | OR (Exp(B)) | p | 95% Wald CI |
| --- | --- | --- | --- |
| BIS-8-S | 1.099 | 0.035 | 1.007-1.199 |
| CERQ_ad* | 1.048 | 0.004 | 1.015-1.082 |
| SCS | 0.972 | 0.043 | 0.945-0.999 |

**Notes**: BIS-8-S=Barratt Impulsivity Scale Shortened; SCS=Self-Compassion Scale; CERQ_ad*=Cognitive Emotion Regulation Questionnaire modified adaptive subscale, without items 20 and 23. Model: Χ^2^(3) = 12.529, p = .006

Table S2b. Parameter estimates in the ordinal logistic GLM of NSSI frequency (N = 120, including men patients with BPD)

| Parameter | OR (Exp(B)) | p | 95% Wald CI |
| --- | --- | --- | --- |
| BIS-8-S | 1.095 | 0.029 | 1.009-1.188 |
| CERQ_ad* | 1.039 | 0.015 | 1.007-1.071 |
| SCS | 0.973 | 0.047 | 0.947-1.000 |

**Notes**: BIS-8-S=Barratt Impulsivity Scale Shortened; SCS=Self-Compassion Scale; CERQ_ad*=Cognitive Emotion Regulation Questionnaire modified adaptive subscale, without items 20 and 23. Model: Χ^2^(3) = 11.489, p = 0.009

Table S3. Differences in NSSI frequency between male and female patients with BPD

| **NSSI frequency category** | Men (N = 11) n(%) | Women (N = 109) n(%) |
| --- | --- | --- |
| 1–3 times per year | 6 (54.50%) | 15 (13.80%) |
| Approximately monthly | 3 (27.30%) | 43 (39.45%) |
| Approximately weekly | 1 (9.10%) | 35 (32.11%) |
| Daily or more frequent | 1 (9.10%) | 16 (14.68%) |

**Notes.** Χ^2^(3) = 11.868, Cramer’s V=0.304, p= 0.008.

Because the number of male participants was small (N = 11), these group differences should be interpreted as trends rather than robust statistical effects.

Table S4. Correlations between psychological variables and NSSI frequency (Spearman’s ρ) and number of NSSI methods (Pearson’s r), in women only (N = 109) and the full sample (N = 120)

|  | Women only (N = 109) | | | | Whole sample (N = 120) | | | | |
| --- | --- | --- | --- | --- | --- | --- | --- | --- | --- |
|  | ρ  (freq.) | p | r  (methods) | p | ρ  (freq.) | p | r  (methods) | p |  |
| FFMQ | -0.041 | 0.670 | -0.083 | 0.394 | -0.019 | 0.836 | -0.080 | 0.386 |  |
| FFMQ_o | 0.186 | 0.053 | 0.079 | 0.415 | **0.229** | **0.012** | 0.092 | 0.320 |  |
| FFMQ_d | -0.068 | 0.484 | -0.109 | 0.259 | -0.051 | 0.578 | -0.093 | 0.315 |  |
| FFMQ_a | -0.099 | 0.307 | -0.037 | 0.704 | -0.059 | 0.525 | -0.028 | 0.759 |  |
| FFMQ_nj | -0.103 | 0.286 | -0.149 | 0.122 | -0.096 | 0.297 | -0.152 | 0.097 |  |
| FFMQ_nr | -0.010 | 0.921 | 0.048 | 0.617 | -0.091 | 0.323 | 0.004 | 0.968 |  |
| RSES | -0.040 | 0.682 | 0.002 | 0.982 | -0.105 | 0.255 | -0.025 | 0.783 |  |
| CERQ_ad* | 0.105 | 0.276 | 0.093 | 0.334 | 0.058 | 0.529 | 0.109 | 0.237 |  |
| CERQ_mad | 0.066 | 0.494 | 0.180 | 0.060 | 0.092 | 0.320 | **0.189** | **0.039** |  |
| BDI-S | 0.070 | 0.468 | 0.084 | 0.384 | 0.175 | 0.056 | 0.138 | 0.133 |  |
| BHS-S | -0.063 | 0.518 | 0.026 | 0.785 | -0.001 | 0.994 | 0.049 | 0.592 |  |
| BIS-8-S | 0.174 | 0.071 | 0.141 | 0.145 | **0.185** | **0.044** | 0.144 | 0.117 |  |
| DES | 0.159 | 0.099 | 0.104 | 0.280 | **0.250** | **0.006** | 0.162 | 0.077 |  |
| SCS | -0.090 | 0.357 | 0.024 | 0.809 | -0.126 | 0.176 | 0.011 | 0.902 |  |

**Notes.** FFMQ = Five-Facet Mindfulness Questionnaire, FFMQ_o=FFMQ observing subscale, FFMQ_d=FFMQ describing subscale, FFMQ_a=FFMQ acting with awareness subscale, FFMQ_nj= FFMQ nonjudging subscale, FFMQ_nr=FFMQ nonreactivity subscale, SCS=Self-Compassion Scale, RSES = Rosenberg Self-Esteem Scale, CERQ = Cognitive Emotion Regulation Questionnaire, CERQ_ad*=CERQ modified adaptive subscale, without items 20 and 23, CERQ_mad = CERQ maladaptive strategies subscale, BDI-S=Beck Depression Inventory Shortened, BHS-S= Beck Hopelessness Inventory Shortened, BIS-8=Barratt Impulsivity Scale Shortened, DES=Dissociative Experience Scale.

Bolded correlations indicate statistical significance at p < 0.05.
